# *Caulerpa lentillifera* extract ameliorates insulin resistance and regulates glucose metabolism in C57BL/KsJ-db/db mice via PI3K/AKT signaling pathway in myocytes

**DOI:** 10.1186/s12967-015-0412-5

**Published:** 2015-02-15

**Authors:** Bhesh Raj Sharma, Hyun Jung Kim, Dong Young Rhyu

**Affiliations:** Department of Oriental Medicine Resources and Institute of Korean Medicine Industry, Mokpo National University, 1666 Youngsan-ro, Muan-gun, 534-729 Jeonnam, Republic of Korea; College of Pharmacy, Mokpo National University, 534-729 Jeonnam, Republic of Korea; Department of Environmental and Molecular Toxicology, Oregon State University, 97331 Corvallis, OR USA

**Keywords:** *Caulerpa lentillifera*, Glucose metabolism, PI3K/AKT, Myocytes, db/db Mice, Anti-diabetic effect

## Abstract

**Background:**

Glucose homeostasis is distorted by defects of the PI3K/AKT and AMPK pathways in insulin-sensitive tissues, allowing the accumulation of glucose in the blood. The purpose of this study was to assess the effects and mechanisms by which ethanol extract of *Caulerpa lentillifera* (CLE) regulates glucose metabolism in C57BL/KsJ-db/db (db/db) mice.

**Methods:**

Mice were administered CLE (250 or 500 mg/kg BW) or rosiglitazone (RSG, 10 mg/kg BW) for 6 weeks. Then, oral glucose tolerance test (OGTT) and intraperitoneal insulin tolerance test (IPITT) were performed, and blood glucose was measured in db/db mice. Levels of insulin and insulin resistance factors in plasma, glycogen content in the liver, and IRS, PI3K, AKT, and GLUT4 expressions in skeletal muscles were measured in db/db mice. Glucose uptake and insulin signaling molecules were measured in L6 myocytes, using fluorometry and Western blotting.

**Results:**

CLE significantly decreased fasting blood glucose, glucose level in OGTT and IPITT, plasma insulin, homeostatic model assessment-insulin resistant (HOMA-IR), TNF-α, IL-6, FFA, TG and TC levels, and hepatic glycogen content in db/db mice. CLE significantly increased the activation of IRS, AKT, PI3K, and GLUT4, which are the key effector molecules of the PI3K/AKT pathway in L6 myocytes and the skeletal muscles of db/db mice. The enhanced glucose uptake by CLE was abolished by treatment with a PI3K inhibitor (LY294002), but not by an AMPK inhibitor (compound C) in L6 myocytes. CLE regulated glucose uptake and homeostasis via the PI3K/AKT pathway in myocytes and db/db mice, respectively.

**Conclusion:**

Our results suggest that CLE could be a potential candidate for the prevention of diabetes.

## Background

Diabetes mellitus is a metabolic disease which is caused by impaired insulin production and/or decreased tissue response to the insulin, and is characterized by elevated blood glucose [1]. The number of people living with diabetes has been increasing worldwide. The International Diabetes Federation estimates that there are 387 million people living with diabetes, and the number is expected to reach 592 million by 2035 [2]. In diabetes, metabolically active cells cannot utilize glucose, so it accumulates in the blood [3]. Glucose is used in target tissues mainly by two pathways, phosphatidylinositol-3-kinase (PI3-kinase) and 5′-AMP-activated kinase (AMPK) [4]. The PI3K/AKT pathway promotes GLUT4 expression in myocytes and adipocytes, and plays an important role in mobilizing glucose throughout the body [5]. The activation of AMPK through pharmacological agents, such as metformin, or by physical exercise, effectively promotes glucose utilization and insulin sensitivity in the body [6]. Thus, the PI3K/AKT and AMPK pathways may be potential targets for the regulation of glucose metabolism in type 2 diabetes and obesity associated with insulin resistance. Guo et al. reported that natural products enhance glucose uptake and decrease insulin resistance by the activation of PI3K/AKT or AMPK in diabetic models [7]. Therefore, interest in the possible therapeutic use of natural products for diabetes has been growing because of the fewer adverse effects comparing to synthetic drugs.

New chemical compounds with various biological activities have been isolated from marine algae [8]. *Caulerpa lentillifera,* a naturally distributed algae in tropical and subtropical regions, has a grape-like appearance, and is used in salads as a vegetable in Japan, Korea, the Philippines, and other countries in south-east Asia [9]. Recently, it has gained popularity in international food markets and the seafood industry because it contains high minerals, dietary fiber, vitamin A, vitamin C, and several essential unsaturated fatty acids [10]. It has been used traditionally to treat high blood pressure, diabetes, rheumatism, and bacterial and fungal infections in the Philippines. Recently, anti-oxidative [9], anti-cancer [11], and lipid-lowering [12] activities have been reported. However, to our knowledge, there is no previous report of its anti-diabetic effects and mechanisms in an animal model of type 2 diabetes. We previously reported *in-vitro* anti-diabetic effects of the ethanol extract of *C. lentillifera* (CLE) [13] and in this paper we report the effects of CLE on insulin resistance and glucose metabolism in db/db mice, and the efficacy was compared with an oral anti-diabetic agent, rosiglitazone (RSG) [14]. Moreover, we explored the mechanism by which glucose was metabolized, using L6 myocytes and db/db mice.

## Materials and Methods

### Reagents

MTT [3-(4,5-dimethylthiazol-2-yl)-2,5-diphenyltetrazolium bromide], dimethyl sulfoxide (DMSO), mercaptoethanol, and an anti-β-actin antibody were purchased from Sigma (St. Louis, MO, USA). 2-[N-(7-nitrobenz-2-oxa-1, 3-diazol-4-yl) amino]-2-deoxyglucose (2-NBDG) was purchased from Invitrogen (Carlsbad, CA, USA). The antibodies to p-IRS-1, IRS, p-AKT, AKT, p-PI3K, PI3K, p-GSK3β, GSK3β, p-AMPK, AMPK, and GLUT4 were purchased from Cell Signaling Technology (Beverly, MA, USA). Nitrocellulose membranes and chemiluminescent reagents for Western blotting were purchased from Bio-Rad (Richmond, CA, USA) and Imegenex (San Diego, CA, USA), respectively. All solvents, chemicals, and reagents were analytical grade and were purchased from Sigma-Aldrich unless otherwise specified.

### Plant material

*C. lentillifera*, purchased from Okinawa, Japan, was extensively washed with tap water, and then kept at room temperature for 30 min to remove the excess water. The taxonomic identity of the plant was confirmed by Prof. Chan Seon Park and the sample was preserved for reference in the herbarium of the Dept. of Oriental Medicine Resources, Mokpo National University, South Korea. The dried *C. lentillifera* (1.7 kg) was extracted with ethanol at room temperature for five times until the color disappeared. The extract was then filtered and evaporated under vacuum, which was followed by freeze drying. The yield of the extract was 1% of the starting material.

### *In-vitro* assay

#### Cell culture and differentiation

L6 cells, a rat skeletal muscle cell line, purchased from the Korean cell bank, were maintained in DMEM supplemented with 10% heat-inactivated FBS. For differentiation into myotubes, cells were seeded in 6-well plates (for immunoblotting) and black 96-well plates (for glucose uptake). After 48 h (>80% confluence), the medium was switched to DMEM with 2% (v/v) horse serum and was replaced after 2 and 4 days, counting the day of differentiation as day 0. Experiments were initiated on day 5, when the myotubes had differentiated fully.

#### Cell viability

L6 cells were seeded at a density of 4 × 10^3^ cells/well in 96-well plates and cultured with or without CLE for 24 h. Briefly, MTT solution (final concentration, 1 mg/mL) was added to each well, followed by incubation at 37°C for 1 h. Finally, DMSO was added to dissolve the formazan crystals. The optical density was then measured at 540 nm using a spectrophotometer (Immuno Mini NJ-2300, Japan).

### Glucose uptake assay

Glucose uptake assay was performed by measuring the uptake of 2-NBDG in differentiated L6 cells. Differentiated cells were rinsed twice with PBS (37°C) and then starved in a serum free DMEM for 3 h. Subsequently, cells were washed twice with PBS (37°C) and then starved for glucose in KRPH buffer (136 mM NaCl, 20 mM HEPES, 1 mM MgSO_4_, 4.7 mM KCl, 5 mM KH_2_PO_4_, and 1 mM CaCl_2_). Then, cells were treated with the sample for 1 h, followed by the addition of 150 μg/mL 2-NBDG, dissolved in PBS, for 30 min. Cells were then washed two times with PBS to remove excess fluorescence in the wells. Then, fluorescence retained by the cells was measured using the fluorescence microplate reader, Perkin Elmer Victor3V 1420 Multilable Plate Counter (Perkin Elmer, USA) at an excitation and emission wavelength of 485 nm and 535 nm, respectively.

### Western blotting

L6 cells were lysed in ice-cold lysis buffer (PRO-PREP; iNtRON Biotechnology, Korea) for 10 min to extract proteins. Skeletal muscle (50 mg) was homogenized in ice-cold lysis buffer (1 mL) to extract proteins. Then, lysed homogenates were centrifuged (13,000 rpm, 20 min, 4°C), and the supernatant was collected as the lysate. The protein concentration of the lysate was determined using the Bio-Rad Protein Assay Agent. Proteins in cell homogenates (30 μg) and tissue homogenates (80 μg) were separated by 10% SDS-PAGE. It was then transferred to the nitrocellulose sheet in western blot apparatus (Bio-Rad, Hercules, CA), and subsequently subjected to immunoblotting using specific antibodies. Finally, the expressed proteins were measured by analyzing the signal captured on the nitrocellulose membrane using an image acquisition and analysis software [15].

### *In-vivo* assay

#### Animals and diets

Six-week-old male C57BL/KsJ-db/db (db/db) mice and their lean heterozygote littermates (db/^+^) were purchased from Central Lab Animal Inc. (Seoul, South Korea), and were maintained under standard living conditions (room temperature of 25°C, 45-50% relative humidity and 12/12-h dark/light cycle) in the Animal Research Center, Mokpo National University. All procedures were approved by the animal ethics committee of Mokpo National University. Despite the plant is edible, we performed acute toxicity studies. As per the expectation, no death was observed up to 2 g/kg BW. Then, we have performed a preliminary assay with a series of dosages of the plant extract (200–1000 mg/kg) on experimental mice. On the basis of observed hypoglycemic effects, we have chosen the doses for this experiment. The 28 db/db mice and 7 db/^+^ mice were fed a pelletized commercial chow diet for 1 week after arrival, then the db/db mice were divided into four groups (*n* = 7 each) based on their blood glucose levels: Control, CLE-250, CLE-500, and RSG-10. Then, the db/^+^ group and control db/db mice were fed a standard semi-synthetic diet (AIN-76), while the other three groups were fed AIN-76 diet with the extract (250 and 500 mg/kg) or RSG (10 mg/kg BW) for 6 weeks. The mice had access to food and water ad libitum. At the end of the experiment, mice were fasted overnight and sacrificed by cervical dislocation. Blood samples were taken from the cardiac puncture to measure plasma bio-markers. Also, the liver, epididymal fat, and skeletal muscle were removed, rinsed with physiological saline solution, and immediately stored at −70°C for further analysis.

### Fasting blood glucose, oral glucose tolerance test (OGTT), and intraperitoneal insulin tolerance test (IPITT)

The blood glucose level was monitored every week after overnight fasting, from the tail vein using a blood glucose test meter. Five weeks after feeding the plant extract, OGTT and IPITT were performed in overnight-fasted mice. For OGTT, all mice were orally administered glucose (2 g/kg body weight) and for IPITT, all mice were injected with insulin (2 units/kg body weight). The blood glucose levels were measured from the tail vein at 0 (prior to glucose or insulin administration), 30, 60, 90, and 120 min after the glucose and insulin loading.

### Biochemical analysis

Blood was collected in a heparin-coated tube and centrifuged (3000 rpm, 20 min, 4°C). Levels of plasma triglycerides (TG) and total cholesterol (TC) were measured spectrophotometrically using commercially available kits (Asan Pharmaceutical Company, Seoul, Korea). The plasma free fatty acid (FFA) concentration was measured using an enzymatic non-esterified fatty acid kit (Zen-Bio, Research Triangle Park, NC, USA). The levels of plasma insulin (ALPCO Diagnostics, Salem, NH, USA), tumor necrosis factor (TNF)-α (Invitrogen, CA, USA), and interleukin (IL)-6 (Thermo Scientific IL, USA) were determined using radioimmunoassay kits. Homeostasis model assessment for insulin resistance (HOMA-IR) was calculated by the following equation [16].

$$ \mathrm{HOMA}-\mathrm{I}\mathrm{R} = \Big[\left(\mathrm{glucose}{-}_{\mathrm{fasting}}\left(\mathrm{m}\mathrm{M}\right) \times \mathrm{insulin}{-}_{\mathrm{fasting}}\left(\mathrm{m}\mathrm{U}/\mathrm{L}\right)\ /\ 22.5\right] $$

### Reverse transcription-polymerase chain reaction (RT-PCR)

Total RNA was isolated from tissues using the TRI reagent (Molecular Research Center, Inc.). Isolated RNA was quantified by measuring OD at 260 and 280 nm using a nanodrop 2000 spectrophotomer (Thermo Scientific, USA). Isolated RNA (50 ng) was added to a final 30 μL volume of Diastar 2 × One-Step RT-PCR premix with forward and reverse primers. The temperature cycle for the PCR reaction was 50°C for 30 min, 95°C for 15 min and 35 cycles of denaturation at 95°C for 20 s, annealing at the respective annealing temperature for 40 s, and extension at 72°C for 1 min, followed by a final extension at 72°C for 5 min. The PCR products were analyzed on a Red Safe (iNtRON Biotechnology, Seoul, Korea)-stained agarose (1.5%) gel, using UVP (image acquisition and analysis software, VisionWork LS). The primers used in RT-PCR are shown in Table 1.

**Table 1:** **Primers for RT-PCR**

**Description**	**Gene bank**	**Sense primer (5′ → 3′)**	**Anti-sense primer (5′ → 3′)**
GCK	NM_009204.2	CCTGCCCGAAAGAGTCTAAAGC	ACTAAGAGCACCGAGACCAACG
G-6Pase	NM_010292.5	TTCACCTTCTCCTTCCCTGTAAGG	TACCAGCTTGAGCAGCACAAGTCG
PEPCK	NM_008061.3	AAGACTCCCAGGACTGGTTCAT	TAGCAGGTAGAATCCAAGCGCG
GLUT4	NM_009204.2	CCTGCCCGAAAGAGTCTAAAGC	ACTAAGAGCACCGAGACCAACG
β-actin	NM_009609.2	TGCCCATCTATGAGGGTTACG	TAGAAGCATTTGCGGTGCACG

### Hepatic glycogen content

Hepatic glycogen content was measured as described previously [17]. Hepatic tissues were homogenized in hot ethanol (80% ethanol) at a tissue concentration of 100 mg/mL, and then centrifuged (9500 rpm, 20 min). The residue was collected, dried over a water bath and then extracted at 0°C for 20 min by adding a mixture of 5 mL water and 6 mL of 52% perchloric acid. The collected material was centrifuged at 9500 rpm for 15 min to recover the supernatant. Next, 0.2 mL supernatant was transferred to a graduated test tube and made to 1 mL by the addition of distilled water. Then, 4 mL anthrone reagent was added to the test tubes and the tubes were then heated in a boiling water bath for 8 min, allowed to cool at room temperature and the intensity of the green to dark-green color of the solution was measured at 630 nm. Glycogen content of the sample was determined from a standard curve prepared with a standard glucose solution.

### Data analysis

Statistical analyses were conducted using SPSS software (SPSS Inc., Chicago, IL, USA). Data are presented as means ± SE. Statistical significance was calculated using one-way analysis of variance followed by Duncan’s *post hoc* comparisons. Differences were considered statistically significant if *p* < 0.05.

## Results

### Effect of CLE on fasting blood glucose and the oral glucose and intraperitoneal insulin tolerance tests in db/db mice

**Figure 1:**
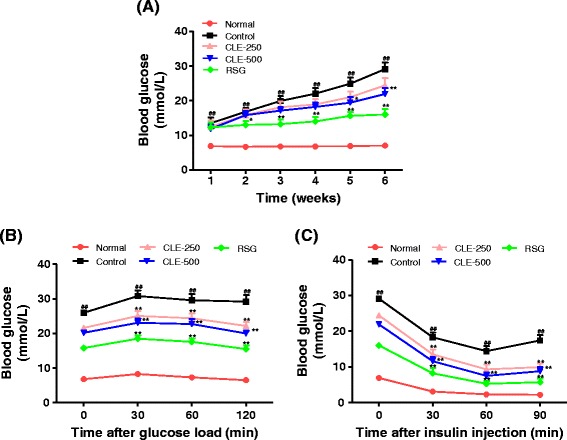
**Effect of CLE on weekly fasting blood glucose (A), and oral glucose (B) and intraperitoneal insulin (C) tolerance test in db/db mice.** For glucose and insulin tolerance tests, after 12 h of fasting, mice were given glucose (2 g/kg body weight) for OGTT and insulin (2 units/kg body weight) for IPITT. The blood glucose level was measured at the indicated times and is presented as mmol/L. Each value represents the mean ± SE, *n* = 7 mice. ^##^ *p* < 0.01, control group *versus* normal group; ^*^ *p* < 0.05 and ^**^ *p* < 0.01, control group *versus* sample treated groups. CLE-250, db/db mice treated with *C. lentillifera* extract at 250 mg/kg body weight, CLE-500, db/db mice treated with *C. lentillifera* extract at 500 mg/kg body weight, RSG-10, db/db mice treated with rosiglitazone at 10 mg/kg body weight.

As shown in Figure 1A, db/db mice showed a continuous increase in blood glucose throughout the experiment. However, the CLE-treated group tended to show lower glucose levels during the experiment. In the final week of the experiment, the CLE-500 group showed blood glucose decreased by 25%, and the RSG-10 group showed blood glucose decreased by 44%, compared with the control group. To evaluate the effects of CLE on insulin resistance in db/db mice, we measured OGTT and IPITT after 5 weeks of treatment. The effect of CLE on OGTT and IPITT is shown in Figure 1B and C. In OGTT, the blood glucose level did not change significantly up to 60 min. However, at 120 min after the glucose load, it was significantly decreased in the normal and CLE- or RSG-administered group, versus the control group. The blood glucose levels in the control group failed to return to baseline after 120 min. Similarly, CLE- or RSG-treated group showed a significant difference in the rapid removal of blood glucose at 60 min versus the control group in IPITT. The normal group showed the most effective glucose removal throughout the experiments.

### Effect of CLE on diet intake, body weight, and weight of epididymal adipose tissue, soleus muscles, and liver in db/db mice

The control group significantly showed higher diet intake, body weight, and the weights of liver and adipose tissues than normal group, whereas the muscle weight in the control group was lower (Table 2). The administration of CLE had no effect on dietary intake, body weight, liver weight, and epididymal fat weight, but significantly increased the muscle weight, by 27.2%, compared with control group. The RSG-treated group, however, showed significant increase in diet intake, body weight, liver, and epididymal fat weight compared with the control group.

**Table 2:** **Effect of CLE on diet intake, body weight, and different organs weights in db/db mice**

**Groups**	**Body weight (g)/mice**	**Diet intake (g)/mice**	**Liver (g)/mice**	**Muscle (g)/mice**	**Epididymal fat (g)/mice**
Normal	27.3 ± 0.6	3.2 ± 0.2	1.01 ± 0.08	0.38 ± 0.02	1.08 ± 0.07
Control	46.0 ± 0.8^##^	5.2 ± 1.8^##^	3.23 ± 0.36^##^	0.16 ± 0.01^##^	1.92 ± 0.09^##^
CLE-250	44.3 ± 1.6	5.1 ± 0.2	2.98 ± 0.19	0.19 ± 0.01^*^	1.84 ± 0.12
CLE-500	45.2 ± 1.6	5.2 ± 0.3	3.21 ± 0.17	0.22 ± 0.01^**^	1.86 ± 0.03
RSG-10	53.3 ± 1.5^**^	6.2 ± 0.2	4.13 ± 0.11^**^	0.22 ± 0.01^**^	2.21 ± 0.05^*^

Each value represents the mean ± SE, *n* = 7 mice. ^##^ *p* < 0.01, control group *versus* normal group; ^*^ *p* < 0.05 and ^**^ *p* < 0.01, control group *versus* sample treated groups. CLE-250, db/db mice treated with *C. lentillifera* extract at 250 mg/kg body weight, CLE-500, db/db mice treated with *C. lentillifera* extract at 500 mg/kg body weight, RSG-10, db/db mice treated with rosiglitazone at 10 mg/kg body weight.

### Effect of CLE on insulin-signaling molecules' expression in the skeletal muscles of db/db mice

**Figure 2:**
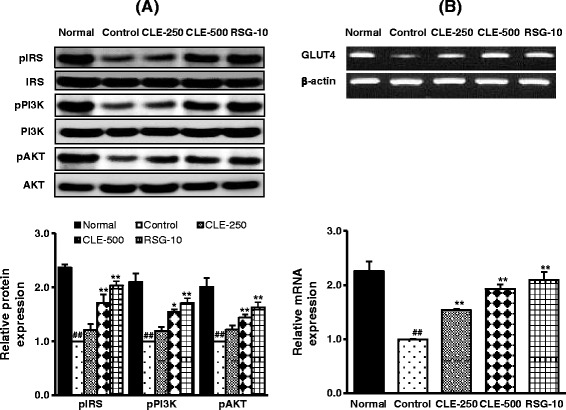
**Effect of CLE on insulin-signaling molecules’ expression in the skeletal muscles of db/db mice.** Following an overnight fasting, mice were sacrificed and the skeletal muscle tissues were collected for mRNA or protein expression of insulin signaling molecules using RT-PCR or Western blot, respectively. **A)** Protein expression; **B)** mRNA expression. Each value represents the mean ± SE, *n* = 7 mice. ^##^ *p* < 0.01, control group *versus* normal group; ^*^ *p* < 0.05 and ^**^ *p* < 0.01, control group *versus* sample treated groups.

As shown in Figure 2A, phosphorylated forms of insulin-signaling molecules, such as IRS, PI3K, and AKT, were significantly decreased in the muscles of control group, compared with the normal group. However, oral administration of CLE for 6 weeks significantly increased the level of those proteins in a dose-dependent manner. Moreover, GLUT4 mRNA expression was also highly expressed in CLE-administered group, compared with control group (Figure 2B).

### Effect of CLE on plasma insulin, HOMA-IR, and markers of insulin resistance in plasma of db/db mice

Plasma insulin, HOMO-IR, TG, and TC levels in CLE-treated group were significantly decreased in a dose-dependent manner compared with control group (Table 3). Similarly, inflammatory markers, such as TNF-α, IL-6, and FFA, which are the root causes of insulin resistance in diabetic patients, were greatly increased in control group, compared with the normal group. However, the oral administration of CLE significantly decreased the level of all those inflammatory markers in a dose-dependent manner. The RSG-treated group was more effective in decreasing inflammatory markers than CLE-treated group.

**Table 3:** **Effect of CLE on plasma insulin, TNF-α, IL-6, FFA, TG, TC, and HOMA-IR in db/db mice**

**Groups**	**Insulin (pmol/mL)**	**HOMA-IR (mM × mU/mL)**	**TNF-α (pg/mL)**	**IL-6 (pg/mL)**	**FFA (mmol/L)**	**TG (mmol/L)**	**TC (mmol/L)**
Normal	16.7 ± 2.7	0.7 ± 0.1	5.0 ± 0.5	25.4 ± 5. 9	0.59 ± 0.08	67.6 ± 7.7	112.2 ± 9.5
Control	121.2 ± 7.8^##^	22.4 ± 2.4^##^	25.0 ± 3.0^##^	100.4 ± 6.3^##^	1.61 ± 0.21^##^	185.7 ± 19.8^##^	229.5 ± 15.2^##^
CLE-250	101.9 ± 8.8	15.1 ± 0.9^**^	19.8 ± 3.6	80.4 ± 6.8	1.40 ± 0.13	140.6 ± 14.6^*^	191.9 ± 17.1
CLE-500	92.3 ± 7.4^*^	12.5 ± 1.1^**^	15.3 ± 2.1^*^	70.4 ± 7.0^*^	1.10 ± 0.15^*^	129.5 ± 16.3^*^	173.3 ± 14.4^*^
RSG-10	75.6 ± 10.0^**^	4.0 ± 0.5^**^	13.0 ± 2.3^**^	55.8 ± 15.0^**^	1.00 ± 0.07^**^	111.9 ± 14.9^**^	155.2 ± 12.0^**^

Each value represents the mean ± SE, *n* = 7 mice. ^##^ *p* < 0.01, control group *versus* normal group; ^*^ *p* < 0.05 and ^**^ *p* < 0.01, control group *versus* sample treated groups. CLE-250, db/db mice treated with *C. lentillifera* extract at 250 mg/kg body weight, CLE-500, db/db mice treated with *C. lentillifera* extract at 500 mg/kg body weight, RSG-10, db/db mice treated with rosiglitazone at 10 mg/kg body weight.

### Effect of CLE on hepatic glycogen content and mRNA expression of hepatic glucose-regulating enzymes in db/db mice

**Figure 3:**
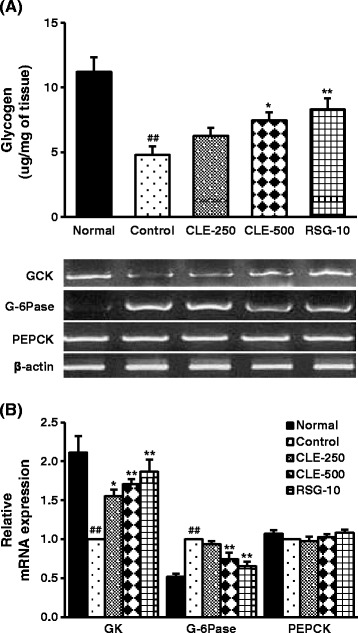
**Effect of CLE on hepatic glycogen and mRNA expression of hepatic glucose-regulating enzymes in db/db mice.** Following an overnight fasting, mice were sacrificed and the hepatic tissues were collected for hepatic glycogen determination, and mRNA expression analysis of hepatic glucose regulating enzymes. **A)** Hepatic glycogen; **B)** mRNA expression. Each value represents the mean ± SE, *n* = 7 mice. ^##^ *p* < 0.01, control group *versus* normal group; ^*^ *p* < 0.05 and ^**^ *p* < 0.01, control group *versus* sample treated groups.

Hepatic glycogen content was significantly decreased in control group, versus the normal group (Figure 3A). CLE-treated group showed an increased hepatic glycogen content. The CLE-500 group had hepatic glycogen increased by 54.8%, whereas RSG-treated group showed hepatic glycogen content increased by 72.7% versus the control group. Hepatic glucose-regulating enzymes glucokinase (GK) and glucose 6-phosphatase (G-6Pase) were significantly decreased and increased, respectively, in the control group versus the normal group (Figure 3B). However, the CLE-500 group showed significantly increased and decreased levels of GK and G-6phase, respectively, compared with the control group. CLE had no apparent effect on hepatic PEPCK activity.

### Effect of CLE on glucose uptake in L6 myocytes

**Figure 4:**
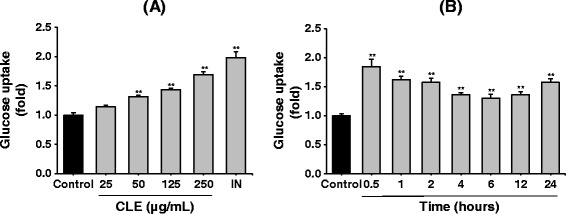
**Effect of CLE on glucose uptake in L6 myocytes.** L6 cells were induced to differentiate with 2% horse serum and treated as indicated. Glucose uptake was first measured at different doses **(A)**. Then, glucose uptake was measured at different time intervals **(B)**, using most effective dose (250 μg/mL). Control: fully differentiated L6 cells. 100 nM insulin (IN) was used as the standard drug. Data are presented as the mean ± SD (*n* = 3). ^*^ *p* < 0.05 and ^**^ *p* < 0.01 *versus* control.

To determine whether CLE could enhance glucose uptake in L6-myocytes, we measured the glucose uptake activity of CLE at different doses (Figure 4A). Then, using the most effective dose (250 μg/mL), glucose uptake was measured at different time intervals (Figure 4B). We found that CLE (250 μg/mL) strongly increased glucose uptake at 30 min post-treatment. Thus, CLE under these optimum conditions, that is 250 μg/mL and 30 min post treatment, was used for further analyses.

### Effect of CLE on insulin-signaling proteins' expression in L6 myocytes

**Figure 5:**
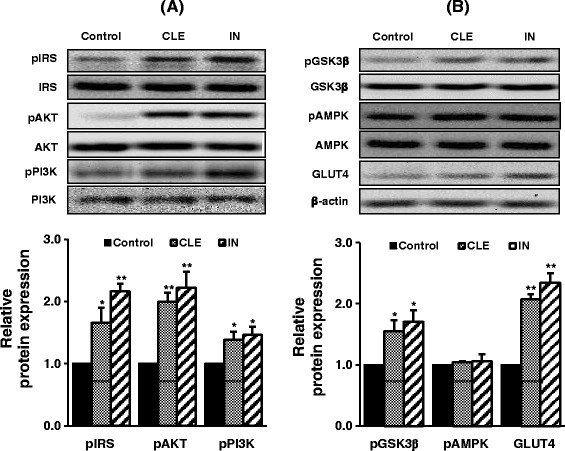
**Effect of CLE on insulin-signaling proteins' expression in L6 myocytes.** Data are representative images for pIRS-1, pAKT, and pPI3K **(A)** and pGSK-3β, pAMPK, and GLUT4 **(B)** from three independent experiments. The scanned bar graph shows the fold induction of control L6 cells for each respective image. L6 cells were induced to differentiate with 2% horse serum and treated as indicated. Control: fully differentiated L6 cells. Data are presented as the mean ± SD (*n* = 3). ^*^ *p* < 0.05 and ^**^ *p* < 0.01 *versus* control.

L6 myocytes were incubated with CLE (250 μg/mL) for 30 min and protein expression of IRS, AKT, PI3K, GSK3β, AMPK, and GLUT4 were measured (Figure 5A and B). CLE significantly enhanced their activation, but CLE did not enhance the phosphorylation of AMPK. The effect of insulin (IN) on the expression of insulin signaling proteins was strong.

### CLE-induced glucose uptake in L6 myocytes is dependent on the PI3K pathway, but not the AMPK pathway

**Figure 6:**
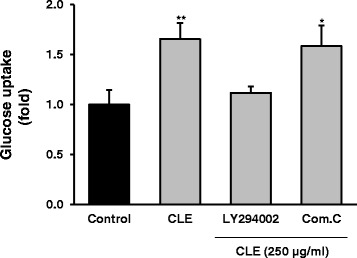
**CLE-induced glucose uptake in fully differentiated L6 myocytes is dependent on the PI3K pathway, but not the AMPK pathway.** L6 cells were treated with CLE with or without compound C (20 μM) or LY294002 (20 μM) for 30 min, and then assayed for glucose uptake using 2-NBDG, a fluorescent derivative of glucose as a glucose analog. Glucose uptake was measured using a fluorescence reader. Data are presented as means ± SE (*n* = 3). ^*^ *p* < 0.05 and ^**^ *p* < 0.01 *versus* control.

To determine the mechanism of glucose uptake, L6 myocytes were incubated with a PI3K inhibitor (LY29002) and an AMPK inhibitor (compound C) with or without CLE for 30 min. As shown in Figure 6, CLE did not stimulate glucose uptake in the presence of the PI3K inhibitor, which blocked the PI3K pathway. However, it did still increase glucose uptake when combined with the AMPK inhibitor, suggesting that CLE-induced glucose uptake is not mediated via the AMPK pathway.

## Discussion

An ideal anti-diabetic drug would improve glucose metabolism and insulin resistance in diabetic patients without causing any side effect. However, currently available anti-diabetic drugs are associated with side effects in long-term use [7]. In the search for safe and effective natural remedies against diabetes, we previously found the effect of CLE on glucose uptake in adipocytes [13]. In this study, we found that CLE decreased blood glucose and improved glucose and insulin tolerance by enhancing glucose uptake via PI3K/AKT signaling pathway in myocytes and db/db mice. Furthermore, CLE increased glycogen content in the liver and decreased inflammatory mediators linked to insulin resistance in the plasma of db/db mice.

Skeletal muscle insulin resistance is a common defect in type 2 diabetes because nearly 90% of the insulin mediated glucose is taken up by skeletal muscle [18]. Both insulin dependent PI3K-signaling and independent AMPK-signaling pathways play important roles in regulating glucose homeostasis via the translation of GLUT4 from the cytoplasm to the plasma membrane [19]. However, defects in these pathways cause insulin resistance in metabolically active tissues, such as skeletal muscle and the liver [20]. As a result, glycogen depletes and glucose output increases in the liver, protein and glycogen breakdown takes place in muscles, and several inflammatory mediators are released into the systemic circulation [21]. In our study, CLE significantly increased the muscle weight, which is directly linked to its glycogen content [22]. Furthermore, CLE increased the activation of insulin-signaling molecules, such as IRS, PI3K, AKT, and GLUT4 in the muscle of db/db mice. CLE was administered once daily for five weeks and then OGTT and IPITT were performed in over-night fasted animals. CLE and RSG treated group showed quick removal of blood glucose. We assumed that maximum effects of CLE and RSG would remain after 12 hours. Herein, we believe that the administration of CLE for five weeks may have activated the glucose utilization machinery in diabetic mice, which would be responsible for the quick removal of blood glucose in both OGTT and IPITT. Our results agree with the recent findings in which glucose-lowering effect of CLE was found in streptozotocin-induced diabetic rats as an animal model of type 1 diabetes [23].

Moreover, the effect of CLE on the activation of insulin signaling molecules in the L6-myocytes was measured using western blotting. Excitingly, as observed in db/db mice, CLE increased the activation of PI3K/AKT signaling molecules such as IRS, PI3K, AKT, GSK3β, and GLUT4 in L6 myocytes. However, CLE showed no apparent effect on AMPK activation in myocytes. To confirm this, we measured glucose uptake activity using AMPK and PI3K inhibitors. As expected, CLE-induced glucose uptake was inhibited only by the PI3K inhibitor, not by the AMPK inhibitor. Thus, we believe that CLE regulated glucose homeostasis and uptake through increasing effects on insulin-dependent PI3K/AKT signaling pathway in myocytes and db/db mice. The glucose uptake activity of CLE is nearly equal to that of 100 nM insulin in our cultured-cells. However, cultured cells are grown in controlled conditions, generally outside of their natural environment, therefore it may be possible that their characteristics can change and may become quite different from those found in the physiological system. By contrast, our *in-vivo* study showed that CLE had mild glucose lowering effects compared with RSG (Figure 1). Animal models are frequently used for research and investigation of human diseases, because animal physiology resembles to that of humans [24]. Therefore, we believe that CLE might have glucose lowering effects in human physiology, as seen in our *in-vivo* assay.

Jung et al. reported that the ethanol extract of the roots of *Brassica rapa* increased hepatic glycogen by regulating glucose sensing enzymes in the liver of db/db mice [25]. Consistently, our findings revealed that CLE increased hepatic glycogen content in db/db mice, which may be due to the regulation of GK and G-6Pase activity. GK stimulates phosphorylation of glucose, which is supposed to antagonize gluconeogenesis and stimulate glycogen storage, whereas G-6Pase hydrolyzes glucose-6-phosphate to phosphate and free glucose [26]. However, to find the precise mechanism by which CLE regulated hepatic glucose metabolism, further studies, showing the effect of CLE on glucose utilization and insulin-signaling molecules’ expression in the liver cells would be required. Nevertheless, we speculate that CLE can facilitate the glucose metabolism in the liver.

Recent studies have shown that plasma concentrations of inflammatory mediators, such as IL-6, TNF-α, and FFA, are increased with insulin resistant states in diabetic patients, and play an important role in deregulating glucose homeostasis [27]. Our findings showed that CLE effectively decreased the production of inflammatory mediators, such as IL-6, TNF-α, and FFA in db/db mice, which could be due to decreased HOMA-IR and insulin, constantly improving insulin resistance in target tissues. In a previous study, Matanjun et al*.* reported that supplementation of 5% *C. lentillifera* to high cholesterol fat-diet significantly decreased serum triglyceride and total cholesterol [12]. Although we have used diet induced and insulin resistant type 2 diabetic mice, our results agree with the previous findings and significantly showed the reduction of TG and TC in CLE-treated mice compared with diabetic mice. We speculate that effect of CLE on the elevated TG and TC may be due to enhanced glucose metabolism in the metabolically active tissues. Thus, our results indicate that effect of CLE on glucose homeostasis was attributed to improvement of insulin resistance in type 2 diabetes.

## Conclusions

In summary, we found that CLE improved glucose metabolism in db/db mice, at least in part, by stimulating glucose uptake in the skeletal muscle via the PI3K/AKT signaling pathway without increasing body weight or dietary intake. CLE contains diverse types of chemical constituents, such as polyphenols [9], minerals, dietary fiber, vitamin A, vitamin C, and several essential unsaturated fatty acids [10]. Their anti-diabetic activities have already been reported [28,29]. The synergistic actions of such components could effectively regulate blood glucose metabolism. Based on these findings, we suggest that CLE may be useful as a candidate for the prevention of diabetes. However, further studies are needed to identify the possible active compounds that are responsible for the anti-diabetic properties of CLE, which may provide an opportunity to develop a new class of anti-diabetic drugs.

## Abbreviations

CLE: *Caulerpa lentifera* ethanol extract
OGTT: Oral glucose tolerance test
IPITT: Intraperitoneal insulin tolerance test
GLUT4: Glucose transporter type 4
HOMA-IR: Homeostatic model assessment-insulin resistant
IL-6: Interleukin-6
TNF-α: Tumor necrosis factor-alpha
FFA: Free fatty acid
TG: Triglyceride
TC: Total cholesterol
AMPK: 5′ adenosine monophosphate-activated protein kinase
IRS: Insulin receptor substrate
PI3K: Phosphatidylinositol 3-kinase
GSK3β: Glycogen synthase kinase 3 beta
2-NBDG: 2-[N-(7-nitrobenz-2-oxa-1, 3-diazol-4-yl) amino]-2-deoxyglucose
